# Conditional Generative Adversarial Networks Aided Motion Correction of Dynamic ^18^F-FDG PET Brain Studies

**DOI:** 10.2967/jnumed.120.248856

**Published:** 2021-06-01

**Authors:** Lalith Kumar Shiyam Sundar, David Iommi, Otto Muzik, Zacharias Chalampalakis, Eva-Maria Klebermass, Marius Hienert, Lucas Rischka, Rupert Lanzenberger, Andreas Hahn, Ekaterina Pataraia, Tatjana Traub-Weidinger, Johann Hummel, Thomas Beyer

**Affiliations:** 1 QIMP Team, Center for Medical Physics and Biomedical Engineering, Medical University of Vienna, Vienna, Austria; 2 Department of Pediatrics, Children’s Hospital of Michigan, The Detroit Medical Center, Wayne State University School of Medicine, Detroit, Michigan; 3 Service Hospitalier Frédéric Joliot, CEA, Inserm, CNRS, Univ. Paris Sud, Université Paris Saclay, Orsay, France; 4 Division of Nuclear Medicine, Department of Biomedical Imaging and Image-guided Therapy, Medical University of Vienna, Vienna, Austria; 5 Department of Psychiatry and Psychotherapy, Medical University of Vienna, Vienna, Austria; and; 6 Department of Neurology, Medical University of Vienna, Austria

**Keywords:** ^18^F-FDG brain, deep learning, head-motion correction, absolute quantification, Patlak analysis

## Abstract

This work set out to develop a motion-correction approach aided by conditional generative adversarial network (cGAN) methodology that allows reliable, data-driven determination of involuntary subject motion during dynamic ^18^F-FDG brain studies. **Methods:** Ten healthy volunteers (5 men/5 women; mean age ± SD, 27 ± 7 y; weight, 70 ± 10 kg) underwent a test–retest ^18^F-FDG PET/MRI examination of the brain (*n* = 20). The imaging protocol consisted of a 60-min PET list-mode acquisition contemporaneously acquired with MRI, including MR navigators and a 3-dimensional time-of-flight MR angiography sequence. Arterial blood samples were collected as a reference standard representing the arterial input function (AIF). Training of the cGAN was performed using 70% of the total datasets (*n* = 16, randomly chosen), which was corrected for motion using MR navigators. The resulting cGAN mappings (between individual frames and the reference frame [55–60 min after injection]) were then applied to the test dataset (remaining 30%, *n* = 6), producing artificially generated low-noise images from early high-noise PET frames. These low-noise images were then coregistered to the reference frame, yielding 3-dimensional motion vectors. Performance of cGAN-aided motion correction was assessed by comparing the image-derived input function (IDIF) extracted from a cGAN-aided motion-corrected dynamic sequence with the AIF based on the areas under the curves (AUCs). Moreover, clinical relevance was assessed through direct comparison of the average cerebral metabolic rates of glucose (CMRGlc) values in gray matter calculated using the AIF and the IDIF. **Results:** The absolute percentage difference between AUCs derived using the motion-corrected IDIF and the AIF was (1.2% + 0.9%). The gray matter CMRGlc values determined using these 2 input functions differed by less than 5% (2.4% + 1.7%). **Conclusion:** A fully automated data-driven motion-compensation approach was established and tested for ^18^F-FDG PET brain imaging. cGAN-aided motion correction enables the translation of noninvasive clinical absolute quantification from PET/MR to PET/CT by allowing the accurate determination of motion vectors from the PET data itself.

## Visual Abstract

**Figure:**
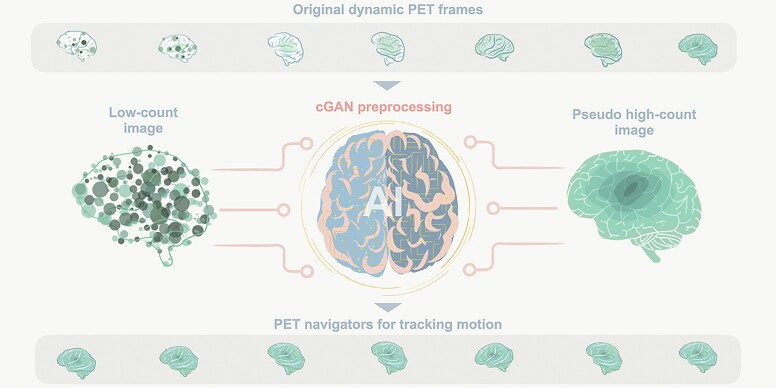

The capability of obtaining fully quantitative physiologic measurements from the human body is a fundamental strength of PET methodology (*1*,*2*). However, due to the requirement of an arterial input function (AIF), the adoption of absolute quantification into clinical work has been severely limited, and only semiquantitative assessments of tracer uptake using standardized uptake expressed as SUV are commonly performed.

In recent years, several methodologies have been proposed to extract an image-derived input function (IDIF) directly from brain PET data to avoid arterial cannulation for determination of an AIF (*3*–*10*). It was demonstrated that a brain IDIF can be calculated either using a combined protocol that integrates PET/CT with MR data (*3*,*7*,*8*) or using a fully integrated PET/MR protocol (*4*,*6*,*9*,*10*). The calculation of an IDIF typically entails, in addition to the definition of a suitable blood-pool region and accounting for partial-volume effects, an accurate correction for involuntary subject motion. A fully integrated PET/MR system is ideally suited to perform all these tasks due to its capability of providing detailed anatomic information, which also includes MR navigators that track motion. However, due to its high cost, the proliferation of PET/MRI into the clinical realm has been severely limited. In contrast, PET/CT is widespread and cost-effective, thus motivating the transfer of IDIF methodology from PET/MR to PET/CT. The definition of a suitable blood-pool region, as well as the geometric correction for partial-volume effects, can be easily accomplished using coregistered PET/CT and MR data. Nonetheless, the accurate correction for subject motion remains a serious challenge in PET/CT imaging.

Assessment of currently available motion-compensation techniques points toward 3 general approaches: data-driven approaches (*11*–*17*), frame-based image registration (*18*,*19*), and real-time hardware motion tracking (*20*). Real-time hardware-based motion tracking detects subject motion with excellent temporal resolution (*20*) but is typically not used in clinical routine due to its complexity and the necessity to integrate external data (motion tracking) with the imaging system (applying the motion vector to images). In contrast, data-driven approaches do not require any external information (such as fiducials or laser positioning system), and they are also less computationally demanding. However, the clinical adoption of frame-based motion-correction schemes has been slow due to poor performance when coregistration is applied to frames that display a dissimilar tracer uptake pattern or noise characteristics as well as the difficulty to correct for intraframe motion in long-duration PET frames (>5 min).

Here, we explored the utility of conditional generative adversarial networks (cGAN) (*21*) as a data-driven approach to facilitate motion correction for involuntary subject motion in dynamic ^18^F-FDG PET studies of the brain. Thereby, we build on recent studies that have shown the potential of cGAN methodology in converting low-count PET images to high-count images (*22*). In general, the objective of cGAN processing is the mapping of a low-count tracer distribution pattern to a high-count pattern based on a priori training data, where generic image features—such as overall brain shape and contours—are likely to be correctly reproduced. Put differently, the creation of high-count images enhances image features that are important for the detection of motion artifacts and as a result might improve the performance of subsequently applied conventional rigid body coregistration routines. It is important to note, however, that the so obtained images are devoid of unique characteristics that are specific for a particular subject and cannot be considered as representing the true (subject-specific) high-count uptake pattern beyond the enhancement of generic image features.

In light of the above, our ultimate objective was to determine the accuracy with which involuntary subject motion occurring during the first part of a dynamic ^18^F-FDG PET/CT study can be detected using conventional motion-correction routines when images are first preprocessed using cGAN methodology, given that early frames are subject to both low-count statistics and dynamically changing tracer uptake patterns. The cGAN preprocessed frames can be thought of as PET navigators whose activity distribution are now temporally invariant, similar to that of the MR navigators. Although our focus was geared toward the derivation of an IDIF, the developed methodology appears to be broader in scope, potentially aiding in improved ability to detect both inter- and intraframe motion. Consequently, our study was guided by the overarching hypothesis that cGAN preprocessing of images can be used to address low-count limitations of short time frame motion-correction strategies and support an accurate data-driven arterial IDIF calculation.

## MATERIALS AND METHODS

Ten healthy volunteers (5 men/5F women; mean age ± SD, 27 ± 7 y) were included in this study (*10*,*11*). The study was approved by the Ethics Committee of the Medical University of Vienna and was performed in accordance with the revised Declaration of Helsinki (1964). All volunteers were deemed to be healthy based on their medical history, physical examinations, and vital signs. Written informed consent was obtained from all subjects before the examinations.

### Study Design

**FIGURE 1.**
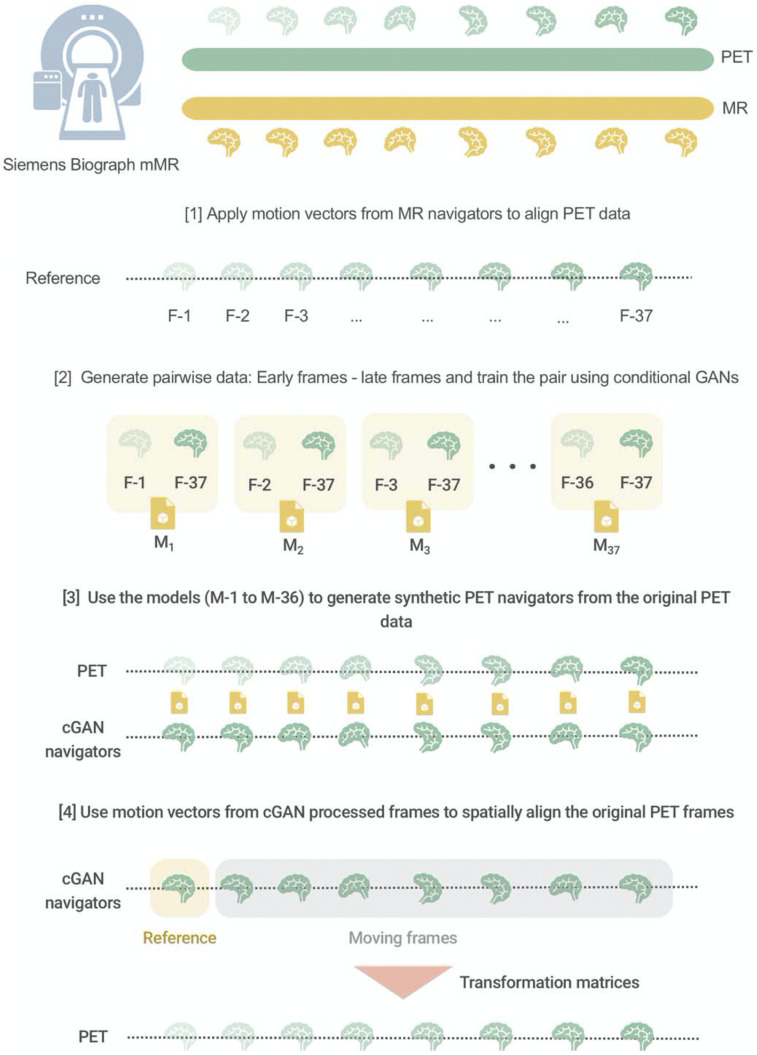
Schematic representation of cGAN methodology for motion correction of dynamic PET frames. [1] Motion vectors from the MR navigators are applied to align the PET data. [2] All training data (*n* = 14) are used to calculate mappings (M_i_, i = 1, 36) between each individual frame (F-1 to F-36) and the reference frame (F-37). [3] These mappings are subsequently used to derive a motion vector based on coregistration of cGAN-produced artificially generated images and the reference image (test data). [4] Application of the motion vector to either rebin frames so that they all correspond spatially or apply the motion vector to the location of region from where a time–activity curve is extracted.

We studied 10 subjects, each of whom underwent 2 PET/MR scans (mean time difference = 17 ± 44 d) in a fully integrated PET/MRI system (Biograph mMR; Siemens Healthineers). To correct the PET study for involuntary subject motion, cGAN image preprocessing was performed before image coregistration, enabling the accurate determination of motion parameters in 3-dimensional (3D) space. These motion parameters were then used to extract the IDIF from the motion-corrected dynamic PET sequence (Fig. 1). To assess the accuracy of the IDIF, arterial blood samples were obtained from a radial artery. Finally, immediately after the PET/MRI examination, a low-dose CT scan of the brain (120 kVp, 50 mAs) was acquired once using a PET/CT system (Biograph TruePoint TrueView 64; Siemens Healthineers, USA) for the purpose of CT-based attenuation correction.

### Imaging Protocol

All examinations were conducted in the afternoon; subjects were asked to keep their eyes open without performing any task. Before each scan, the glucose concentration (mmol/L) in blood was measured, and a venous line was established for the injection of the ^18^F-FDG tracer. In addition, an arterial line was established in the contralateral arm for manual arterial blood sampling. To ensure a high signal-to-noise ratio in the MR images, a head and neck coil was used.

After positioning the brain in the center of the field of view, a 60-min PET list-mode acquisition was initiated with the bolus injection of ^18^F-FDG (352 ± 66 MBq). Contemporaneously with the PET data acquisition, multiple MR sequences were acquired: a 3D time-of-flight MR angiography (TOF-MRA) sequence (0.5 × 0.5 × 1 mm voxel size, echo time = 3.6 ms, repetition time = 21 ms, 25° flip angle, 228 × 384 matrix, 220 slices) for the definition of the carotid vasculature and a T1-weighted MRI sequence (1 × 1 × 1 mm voxel size, 256 × 256 matrix, 192 slices) for anatomic localization. Sparsely sampled MR navigators (2D-EPI, 3.0 × 3.0 × 3.0 mm voxels, 64 × 64 matrix, 36 slices, echo time = 30 ms, repetition time = 3,000 ms) were interleaved at specific time intervals (0, 2.5, 5, 7.5, 10, 14, 17, 21, 26, 33, 38, 42, 44, and 50.5 min after injection), yielding for each time point a 3D image volume (*23*) that allowed the determination of subject motion with 6° of freedom (translation in *x,y,z* direction and rotation with respect to the 3 Euler angles). These 6 motion parameters defined a motion vector, which was used to ensure spatial correspondence between early frames and late frames for cGAN training.

PET list-mode data were rebinned into a dynamic frame sequence (24 × 5 s, 1 × 60 s, 1 × 120 s, 11 × 300 s) and were reconstructed (e7 tools; Siemens) into a 344 × 344 × 127 matrix (voxel size, 2.08 × 2.08 × 2.03 mm) using the ordinary Poisson ordered-subset expectation maximization 3D algorithm (3 iterations, 28 subsets, 2 mm gaussian filter). Attenuation and scatter correction were performed using CT-based attenuation-corrected maps corrected for motion in each PET frame.

### Blood Sampling

Arterial blood samples were collected manually at different time points (24 × 5 s, 1 × 60 s, 1 × 120 s, 1 × 300 s, 1 × 600 s, 2 × 1,200 s after injection) from the radial artery. Whole-blood radioactivity concentrations were measured using a γ-counter (2480 Wizard2 automatic γ-counter; PerkinElmer). To obtain the arterial input function (AIF), whole-blood samples were centrifuged to separate the plasma component, followed by the measurement of radioactivity in the plasma. The measured whole-blood and plasma tracer concentrations were used to calculate the time-dependent plasma–to–whole-blood ratios for each subject.

### 3D-cGAN

Generative Adversarial Networks (GANs) are generative algorithms, which belong to the field of unsupervised learning (*24*). The architecture of a GAN consists of 2 convolutional neural networks that together constitute an opponent-component system: a neural network (termed the Generator *G*) that generates artificial data based on a training dataset, and a neural network (termed the Discriminator *D*) that classifies the artificially created data as being either real (i.e., belonging to the training dataset) or being artificially generated.

Conditional GANs (cGANs) are a supervised extension of the GAN framework (*25*). Although GANs typically perform a mapping operation from a random noise vector (*z*) to an output vector (*y*) expressed as (*G: z → y*), cGANs perform a mapping operation from an observed image (*x*) and a random noise vector (*z*) to an output image (*y*), expressed as (*G: {*x, *z} → y*). Here, the mapping operation is the process of linking 2 image patterns together and is “learned” from a training set that defines the true correspondence between pairs of input and output images (*x→y*). In short, the training dataset provides a general mapping of 2 images with different noise characteristics (e.g., a low-count to a high-count image). It is a generic mapping operation that accounts for broad features in the 2 images but does not account for subject-specific attributes. Accordingly, such mapping is representative for the transformation of any ^18^F-FDG uptake image obtained at a particular time (e.g., 2–3 min after injection) to any ^18^F-FDG uptake image at a later time (e.g., 55–60 min after injection).

In this 3D-cGAN implementation, corresponding pairs of low-count (early) and high-count (late) PET frame images were used to define the mapping operation (*G*) by minimizing a loss function expressed as:

Eq. 1 $$\begin{array}{c} {ん}_{cGAN}\left(G,D\right)=E_{x,y}\left[log\;D\left(x,y\right)\right]+E_{x}\left[log\left(1-D\left(x,G\left(x\right)\right)\right)\right] \end{array},$$

where *G* attempts to minimize the loss function (${ん}_{cGAN}$), whereas *D* strives to maximize it (i.e., G* = arg min_*G*_ *max*_*D*_ ${ん}_{cGAN}$(G, D)). To create artificially generated high-count images from low-count (early) PET frames, we added an estimation error loss to the Discriminator feedback for the effective training of the Generator (*G*) (*23*). The final loss function, $G^{*}$ is then expressed as:

Eq. 2 $$\begin{array}{c} G^{*}\;=\arg min_{G}max_{D\;}{ん}_{cGAN}\left(G,D\right)+\lambda {ん}_{L1}\left(G\right) \end{array},$$

where ${ん}_{L1}\left(G\right)$ is an additional L1-norm based loss function for the generator, and *λ* is a tunable parameter, which is greater than zero (in our case λ=1 (*24*),). The U-netlike architecture (*26*) with skip connections was used as the Generator network (Supplemental Fig. 1; supplemental materials are available at http://jnm.snmjournals.org), taking 3D subvolumes of the original early frame PET image as input. The skip connections facilitate the preservation of the local image information that is lost during the initial down-sampling process and transfer this information to the later occurring up-sampling phase of the network. We used a *PatchGAN* (Supplemental Fig. 2) as a Discriminator (*22*), which classifies each given patch as either true or artificial. In addition, we added 2 more convolutional layers to the Discriminator architecture. The advantage of this architecture is that the network preserves the high-frequency structures of the high-count (late) PET frames using fewer parameters than would be required using the full-size images. Training of the convolutional neural networks was performed using the standard method from Goodfellow et al. (*24*).

### cGAN-Aided Motion Correction

**FIGURE 2.**
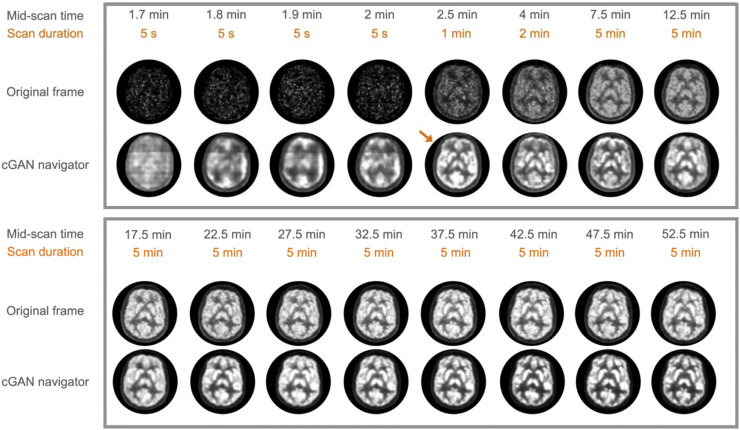
Results of cGAN processing for a representative subject. (Top and bottom panels, first rows) Original PET frames at different mid-scan times (1.7 to 52.5 min) with various frame durations (5 s, 1 min, 2 min, and 5 min). (Top and bottom panels, second rows) Corresponding, artificially generated, high-count PET images. cGAN processing is able to produce a tracer distribution pattern that is similar to the reference frame as early as 2.5 min after injection (arrow, top row).

A random 70%-to-30% data split of the full data was used for cGAN training (14 scans) and testing (6 scans). Initially, all studies in the training set (14 measurements with 37 frames each) were corrected for motion using motion vectors obtained from the contemporaneously acquired MR navigators (*23*) (Fig. 1). Real-time data augmentation (rotation, translation, shearing, additive gaussian noise, brightness, contrast) was performed on the training datasets, resulting in 21,000 synthetic datasets. Subsequently, cGAN mapping was performed between the last high-count PET frame (reference frame 37, representing tracer accumulation at 55–60 min after injection) and all other PET frames, resulting in 36 mappings (x*→y*) with variable quality (Fig. 2).

The obtained mappings were then applied to the test datasets to obtain artificially generated high-count images (using cGAN) that imitate the distribution of the reference frame from the original low-count images. After the generation of the cGAN-based high-count images for the test datasets, motion correction was performed by considering the 55–60 min postinjection. PET frame as reference frame (F_ref_), and all other frames were subsequently registered to the F_ref_ using a standard multiscale mutual information (MI) routine (Greedy module ITK 1.2.4, Insight Segmentation and Registration Toolkit). For each frame of the dynamic sequence, this approach resulted in a 6-parameter motion vector.

### Standard PET Frame–Based Motion Correction

To evaluate the added value of cGAN-aided motion correction, this methodology was compared with a standard PET frame–based motion correction. PET image frames were aligned using the same multiscale MI-based coregistration routine as described above (Greedy module ITK 1.2.4). This routine performs alignment between images starting at a coarse scale, which is then used to initialize registration at the next finer scale, a process repeated until it reaches the finest possible scale. As for the early images (<3 min after injection), the applied multiscale MI coregistration approach failed due to insufficient count statistics, thus we summed the first 3 min of the dynamic sequence to create a reference frame with sufficient statistics. Subsequently, all later frames (>3 min after injection) were rigidly aligned to this summed frame. It is important to point out that this approach (summing of early frames) is frequently implemented in dynamic studies when low-count images that do not contain sufficient data that would allow extraction of an accurate motion vector are analyzed. Because robustness of this coregistration procedure can be improved by low-level smoothing (The ITK Software Guide; Kitware Inc.), our standard registration approach therefore consisted of applying a heuristically chosen 4-mm gaussian filter to the images before registration. However, to assess the performance of cGAN methodology when processing the original (low-count) images, this smoothing step was omitted when testing cGAN-processed images.

### PET Emission Data and Attenuation Map Alignment

To account for misalignment between the PET emission data and the attenuation map, we used a dual-reconstruction approach. Specifically, PET attenuation correction was performed on the basis of an attenuation map derived from a CT image acquired immediately after the PET/MRI protocol. This CT attenuation map was coregistered to the first MR sequence of the study protocol (TOF-MRA sequence), and this static map was then used to perform attenuation correction for the whole PET dynamic sequence. However, this approach does not take into account PET interframe motion. Therefore, non–motion-corrected PET frames were initially used to derive a motion vector (using either MR navigators for the training data or cGAN-processed images for the test data), and once the motion vector was determined this information was used to align the CT attenuation map to each PET dynamic frame. The motion-corrected CT attenuation maps were subsequently used to re-reconstruct the whole dynamic PET sequence.

### Characterization of cGAN Performance

To assess the degree to which cGAN image processing is able to enhance generic features of brain tracer distribution (such as overall brain shape and contours), cGAN performance of individual frames was assessed on the basis of 2 measures: first, by the improvement in MI between the reference image and the cGAN-generated high-count images relative to the original images, and second, by the comparison of the absolute percentage difference with respect to the histogram area-under-the-curve (Hist_AUC_) between histograms derived from cGAN-generated and reference images. It is important to note that these histograms include all image voxels in 3D space and are not affected by subject motion. Moreover, the MI and Hist_AUC_ are complementary; MI is sensitive to the similarity in image patterns expressed in the 2 images, whereas Hist_AUC_ provides information with respect to scale relationship between voxel intensities in the 2 images (*27*,*28*).

### Generation of Simulated Test Datasets

Given the low number of the original test datasets (6 scans), additional test datasets were generated on the basis of the original 6 test datasets. These simulated datasets were used to further investigate the potential of the cGAN method to address the problem of interframe motion.

Excessive interframe motion was simulated by adding to each dynamic frame (except the reference frame) an arbitrary translation (0, 1, or 2 voxels in each direction) and rotation (0°, 0.5°, or 1° for each Euler angle) vector (SimpleITK 1.2.4, Supplemental Fig. 3). Ten repetitions were performed for each dataset with different motion vectors added, resulting in a total of 60 synthetically created test datasets, each consisting of 37 dynamic frames. These datasets were then either preprocessed using cGAN methodology or were directly coregistered to the reference frame (standard PET frame–based motion correction) using the normalized MI alignment routine as described above.

Moreover, to assess the potential of cGAN methodology to aid in the detection of intraframe motion in routine clinical static studies, we partitioned the list-mode data of the reference frames (#37 from 55–60 min after injection) into a series of short subframes (10 subframes of 30 s and 20 subframes of 15 s). cGAN was used to produce artificially generated high-count images from these subframes. This procedure was applied for each measurement of the test dataset (6 scans). Improvement in image quality was assessed on the basis of the increase in MI after cGAN processing. In addition, to further demonstrate the ability of cGAN processing in accurately accounting for intraframe motion, we selected a representative reference frame, which we partitioned into 15-s subframes and introduced random motion (translation 3–5 mm, rotation < 1°) to the subframes. After motion correction either with (cGAN-aided) or without cGAN preprocessing, the coregistered subframes were summed and the resulting images were visually compared for image quality.

### cGAN-Based IDIF

To assess the clinical performance of the cGAN method for motion compensation of dynamic PET frames, we extracted the IDIF from the test dataset (both original and simulated) and compared the IDIF with the AIF. For this, we replaced the MRI navigator-based motion correction in our previously developed IDIF pipeline with the developed cGAN-aided motion correction. This analysis pipeline was described in detail previously (*9*,*10*). In brief, it entails automated segmentation of the petrous region of the internal carotid arteries (ICA) from the corresponding TOF-MRA images followed by an automatic multiscale intermodal NMI coregistration (Greedy ITK 1.2.4) of the TOF-MRA volume and the reference frame (frame #37) for each study. The arterial blood-pool region was defined on the basis of the MR angiography image, clearly identifying the ICA region. This region was subsequently transferred into PET space where it was used to extract the time–activity curve. cGAN-aided motion vectors were applied during the extraction of the time–activity curve to adjust the blood-pool region for the computed displacements. Finally, an iterative regional partial volume correction procedure was applied in each PET frame to recover the true activity in the internal carotid arteries (*10*).

### Postprocessing of IDIF

The motion- and partial volume–corrected IDIF was interpolated with a step length of 1 using a “Piecewise Cubic Hermite Interpolating Polynomial” to match the blood sampling times. All corrections were applied to the IDIF, with the AIF being considered as the reference (*3*,*9*). First, count rates from sampled arterial blood were scaled using the cross-calibration factor between the PET/MR and the on-site γ-counter to express the AIF in the same units as the PET data (Bq/mL). Second, a plasma IDIF was derived on the basis of the individual plasma-to-blood ratios obtained from sampled arterial blood of the study subjects. Third, the delay between the AIF and the IDIF was corrected by shifting the IDIF curve to match the sampling times of the AIF. Finally, due to the difference in sampling location (ICA for IDIF and radial arteries for AIF), a monoexponential dispersion function with a tau value of 5 s (*29*) was convolved with the IDIF to mimic the dispersion effects.

### Quantification of CMRGlc

Calculation of cerebral metabolic rate of glucose (CMRGlc) for the test datasets was performed using a voxelwise Patlak graphical analysis (lumped constant = 0.65 (*30*)) using either the AIF or the IDIF. Analyses were performed using in-house–developed Matlab tools (Matlab R2018a; The MathWorks) that generate parametric images representing CMRGlc (umol/100 g/min). In particular, a linear function was fitted to the Patlak-transformed data, including data from 25 min after injection until the end of the scan (8 data points). The resulting slope was then multiplied with the subject’s plasma glucose level (umol/L) and divided by the lumped constant. By applying a gray matter (GM) mask derived from individually coregistered T1-weighted MR images, the average CMRGlc value in the GM was determined using either the AIF (CMRGlc_AIF_) or the IDIF (CMRGlc_IDIF_).

### Assessment of cGAN Performance for Motion Compensation

The quality of cGAN-aided motion correction of the dynamic frame sequence was assessed in relation to the sampled AIF. Specifically, IDIFs were determined from the test datasets (both original and simulated) using cGAN-aided motion vectors and compared with the AIFs using the area under the curve (AUC). Differences in GM CMRGlc values derived from the IDIF and AIF were assessed using the absolute percentage difference (|%Δ|) between CMRGlc values:

Eq. 3 $$|\text{\%Δ}|=\left|\frac{CMRGlc_{IDIF}-CMRGlc_{AIF}}{CMRGlc_{AIF}}\right|\;x\;100\;.$$

cGAN performance was assessed separately for the original dataset (*n* = 6) and the simulated dataset (*n* = 60).

## RESULTS

Figure 2 visualizes the results of cGAN processing of the dynamic frame sequence from approximately 100 s (1.7 min) after injection until the penultimate frame in the study (#36). The data indicate a visually excellent quality of the artificially generated images for frames of 60 s duration, as early as 2 min after injection of the tracer. In contrast, the quality of cGAN images appears suboptimal for very short frames (5 s) before 2 min after injection.

**FIGURE 3.**
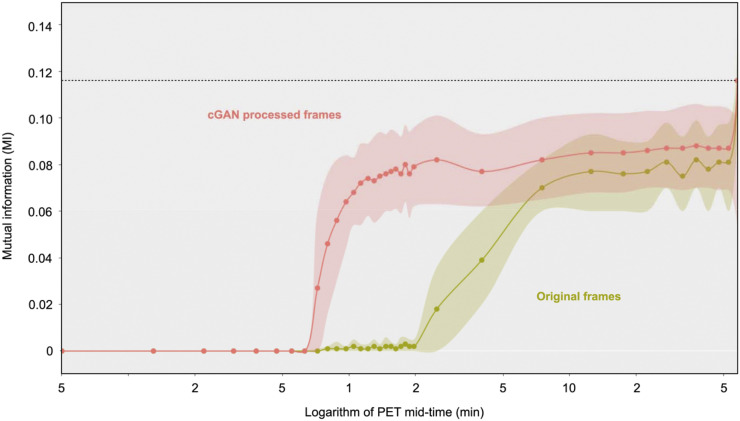
Comparison of MI index between the original (green) and cGAN processed (red) dynamic frames with the reference frame as a (log) function of scan time. Note that neither dynamic sequence is motion corrected. Values represent the average and the SD (shown as shaded area) of the test dataset (*n* = 6). cGAN processing increases MI of ^18^F-FDG brain images with respect to the reference frame close to the MI of late frames (dotted line).

**FIGURE 4.**
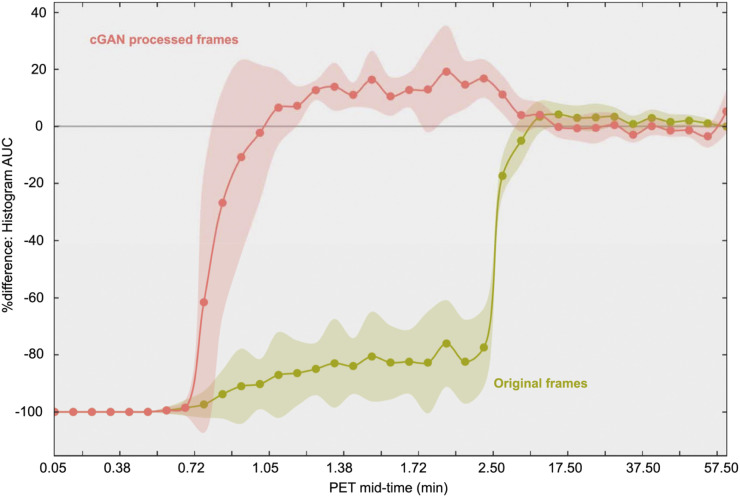
The |%Δ| of the AUC between the reference image histogram and image histograms derived from cGAN-processed images (red) and original images (green) as a function of scan time. Note, images were not motion corrected because histograms are independent from motion. The percentage difference is substantially decreased for low-count (short) frames between 1 min and 2 min after injection, suggesting that an accurate motion vector can be determined even for early frames.

Quantitative assessment of cGAN performance based on MI is depicted in Figure 3. A substantial increase in MI of the individual frames after the application of cGANs, in frames as early as 1 min after injection, is clearly noted. Figure 4 indicates a substantial decrease in the |%| between histogram AUCs characterizing cGAN-processed images and those characterizing the original images during the very early phase of the study (60–120 s). During that time, cGAN-processed images derived from the very short frames (5 s) decreased the difference in histogram AUC by approximately 80% relative to histogram AUCs obtained from the original images. Subsequent improvements were minor (<5%) for longer and later frames with better count statistics.

**FIGURE 5.**
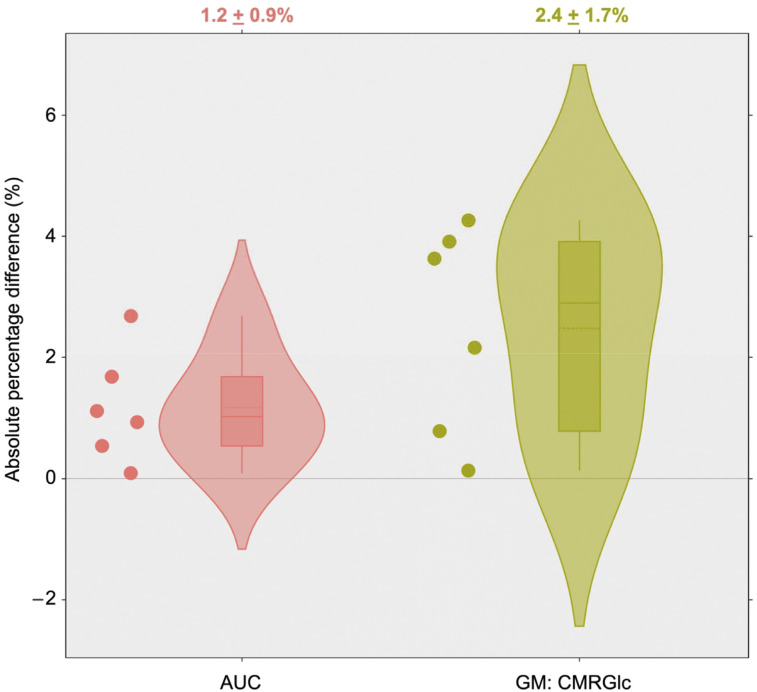
The |%Δ| between AUC and GM CMRGlc values (*n* = 6) obtained using the cGAN-based IDIF and AIF. All differences were within 5% of the AIF standard.

Supplemental Figure 4 demonstrates that cGAN-based IDIFs were closer to the reference standard in comparison to the IDIFs obtained without cGAN processing and using only standard frame-based motion correction (motion profile of the represented subjects in Supplemental Fig. 5). For the original dataset (*n* = 6), the |%Δ| between AUCs derived using the motion-corrected IDIF and the AIF was 1.2% ± 0.9%. The GM CMRGlc values determined using these 2 input functions differed by less than 5% (2.4% ± 1.7%) (Fig. 5). The quantitative difference in AUC and GM CMRGlc values between AIF and IDIF (before and after cGAN preprocessing) for individual datasets (*n* = 6) with their respective augmentations (10 iterations) is depicted in Table 1. For the simulated datasets (*n* = 6 × 10 iterations), the mean difference in AUC values between those obtained using the AIF and the IDIF using cGAN preprocessing was 0.9% ± 0.7%, whereas the difference in AUC values between AIF and the IDIF without cGAN preprocessing was 2.9% ± 3.1%. Moreover, IDIFs extracted from cGAN-preprocessed motion compensated data resulted in CMRGlc values closer to those obtained using the AIF, with an absolute difference of 2.2% ± 1.8% as compared with CMRGlc values determined without cGAN preprocessing of 3.9% ± 3.5%. The improved performance of cGAN-aided as compared with non–cGAN-aided motion correction can be also inferred from the smaller variance of both AUC and GM CMRGlc values in the case of cGAN preprocessing.

**TABLE 1:** The |%Δ| Between AUC or GM CMRGlc Values (*n* = 6 × 10 Synthetic Datasets) Using the IDIF (Motion Corrected With cGAN and Without cGAN Processing) and Values Obtained Using Arterial Blood Sampling

Patient ID	AUC original PET frames (mean ± SD %)	AUC cGAN processed frames (mean ± SD %)	GM CMRGlc original PET frames (mean ± SD %)	GM CMRGlc cGAN processed frames (mean ± SD %)
P-01	1.4 ± 0.8	0.8 ± 0.3	3.8 ± 2.3	3.9 ± 0.4
P-02	2.0 ± 1.4	0.4 ± 0.3	2.7 ± 2.5	1.3 ± 1.0
P-03	8.3 ± 1.7	1.7 ± 0.4	10.1 ± 2.6	0.9 ± 0.6
P-04	4.3 ± 2.9	0.8 ± 0.7	3.1 ± 2.3	0.9 ± 0.8
P-05	1.1 ± 0.6	0.2 ± 0.2	1.9 ± 0.6	1.0 ± 0.3
P-06	1.3 ± 0.6	0.5 ± 0.4	4.9 ± 0.9	1.9 ± 0.6

**FIGURE 6.**
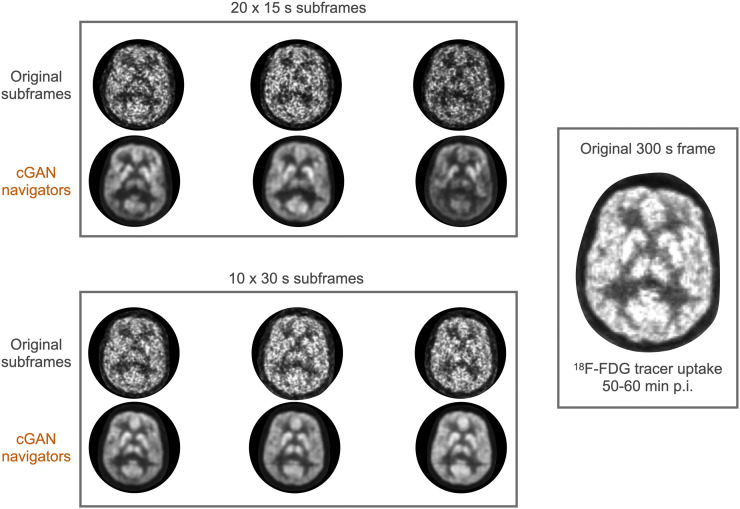
Performance of cGAN processing when applied to a subset of frames that were partitioned into frames of either 15-s duration (left upper panel) or 30-s duration (left lower panel) from an original 300-s static ^18^F-FDG frame at 55 min after injection (right panel). The image quality of the processed subframes for both subsets is substantially improved when compared with the unprocessed subframes and resembles closely the original 300-s frame that includes all data.

**FIGURE 7.**
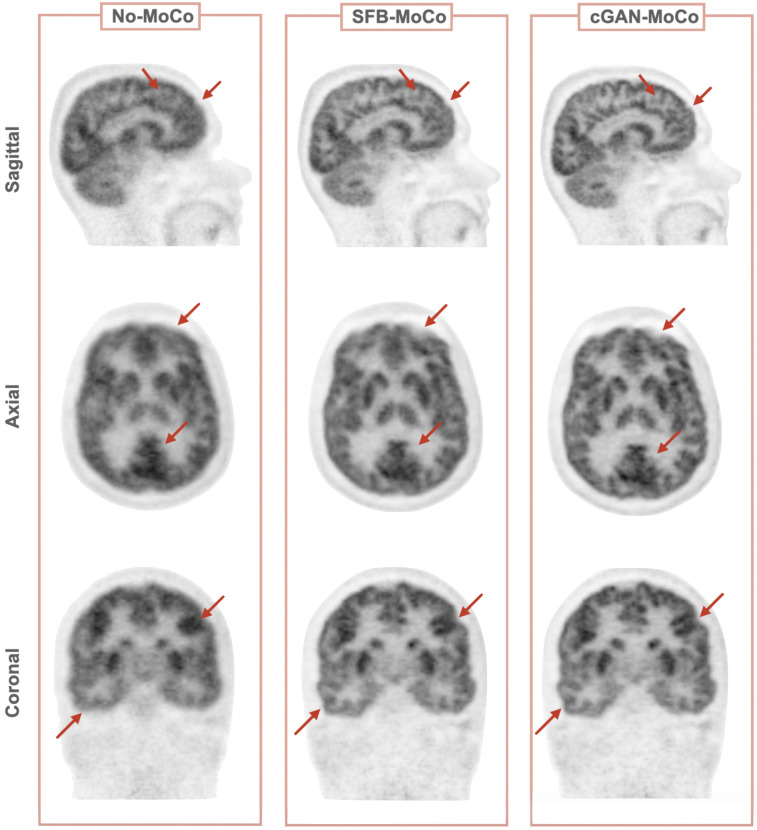
Summed images corresponding to 55–60 min p.i, after intraframe motion correction using motion vectors obtained from 15-s subframes. (Left column) Summed images without correction for intraframe motion (No-MoCo). (Middle column) Summed images after standard frame-based motion correction without cGAN preprocessing (SFB-MoCo). (Right column) Summed images using cGAN-aided motion correction (cGAN-MoCo). Red arrows indicate the areas of improvement.

Figure 6 shows representative images that were obtained by partitioning the reference image (55–60 min after injection) into 15-s subframes and the image quality of these subframes after cGAN processing. For the 10 × 30 s subframe dataset, the MI improved by 135% (from 0.030 ± 0.003 to 0.070 ± 0.001), whereas improvement was even greater for the 20 × 15 s dataset (improvement of 290%; from 0.002 ± 0.003 to 0.0700 ± 0.0001). Moreover, Figure 7 demonstrates the improvement in image quality of the reference image when 15-s subframes with artificially introduced random motion underwent standard frame-based motion correction and cGAN preprocessing before rigid-body motion correction as compared with non–motion-corrected summed subframes. As expected, motion correction improves image sharpness, and one can appreciate a slight improvement of images processed using cGAN methodology as compared with those processed with standard frame-based motion correction.

## DISCUSSION

We present a fully automated motion-correction approach for dynamic ^18^F-FDG PET studies of the brain that uses cGAN preprocessing of low-count images to improve the estimation of motion vectors derived using conventional rigid-body coregistration algorithms (Fig. 2). Our results suggest that cGAN methodology allows the creation of artificially generated high-count ^18^F-FDG brain images from early low-count images that closely resemble the ^18^F-FDG uptake pattern at late (∼60 min after injection) scan times. The creation of artificially generated, high-count images allows then the reliable determination of a motion vector directly from the data, which was verified by the comparison of an IDIF with arterial blood samples. Thus, cGAN-aided motion correction is likely to have a substantial impact on the quality of dynamic low-count ^18^F-FDG PET brain studies and as a result might contribute to the expansion of absolute brain quantification into clinical routine. Especially in the context of clinical PET/CT imaging, cGAN preprocessing of low-count image frames could play an important role in improving the performance of established motion-correction approaches. The developed cGAN methodology also shows promise in addressing the problem of intraframe motion in long-duration (5–20 min) PET scans, by allowing the partitioning of a frame into subframes which, after cGAN preprocessing, can be accurately corrected for motion.

To prevent motion when imaging the brain, subjects are usually instructed to remain motionless, and their head is immobilized using bands that affix the skull to the head rest (*31*). This approach works reasonably well when imaging cooperative subjects, but frequently fails in the clinical population due to the subjects being either uncomfortable or claustrophobic within the PET gantry (*31*). As a result, motion artifacts are frequently encountered in both static and dynamic PET imaging. The problem with motion artifacts is even more severe when an IDIF is extracted from the dynamic PET sequence, given that the magnitude of random displacements and the system resolution are typically larger than the small size of the arteries. Accordingly, accurate motion correction is an important prerequisite for absolute quantification in PET imaging (*17*).

It has been well recognized that the original low-count/high-noise images are poorly suited for alignment due to the poor definition of image landmarks that could guide the registration procedure. As such, low-count images require some form of preprocessing to achieve a satisfactory performance of the subsequently applied coregistration routines (*32*). This preprocessing step could include various forms of smoothing or morphologic operations, but could also consist of more sophisticated forms of processing, such as the cGAN methodology. From a conceptual point of view, cGAN preprocessing might be superior to the previously applied methods, as cGAN processing is based on an automated (i.e., operator-independent) mapping of low-count images to their true high-count match. Stated differently, the calculated mapping is specific to the noise characteristics of the original low-count images, and the resulting artificially generated images represent the most likely prediction of the final (high-count) tracer distribution one could expect based on the training data. Overall, our data clearly highlight the strengths of cGAN processing, such as the autonomous optimized smoothing and “smart” inpainting, which substantially enhance the information content of low-count images so that coregistration algorithms are provided with sufficient information to accurately estimate the motion parameters.

In practical terms, the functionality of cGANs consists in the ability to correctly predict the local distribution of measured data based on a statistically insufficient sample. More specifically, the cGAN algorithm extracts the most likely relationships between low-count images (where the underlying distribution is ambiguous) and high-count images (where the underlying distribution is well defined) from the training dataset and applies the extracted relationships to new images. As a result, cGAN methodology is able to accurately predict generic image features (such as brain contours) from low-count images. The improved definition of brain contours then allows improved performance of conventional MI coregistration routines that strongly depend on well-defined imaging features.

Images with dissimilar uptake pattern are typically encountered in dynamic studies when the tracer uptake pattern changes as a function of time during the frame sequence. Our results showed that structural information inherent to very early low-count images (<2 min after injection) is insufficient to generate an acceptable mapping with the reference frame, thus precluding the generation of a high-count image that could guide the derivation of an accurate motion vector. Conversely, ^18^F-FDG brain uptake at times > 2 min after injection appears to be sufficient for adequate cGAN mapping if the frames are not too short (>30 s). However, we acknowledge that the relevance of cGAN processing is strongly diminished in the case of high-count/low-noise images that are already characterized by well-defined features. Finally, despite the fact that histograms derived from cGAN-processed images have a similar overall shape with respect to the reference images (Supplemental Fig. 6), they tend to overrepresent high intensities and should not be used in lieu of the original low-count images for clinical diagnosis.

Data from our previous studies suggest that motion magnitude increases with time, and for very early time points (<2 min) the motion magnitude tends to be negligible (i.e., translation all axes < 1 mm, rotation <1° in all axes) (*9*,*10*). Moreover, even in the case of subject movement in this very early phase of the study, the error accrued in the integral under the blood time–activity curve remains negligible. We demonstrated this accuracy in our own test dataset, showing a mean value of <1.5% for the absolute difference between the IDIF and the sampled arterial blood curve, which translated to an average difference of <3% for the calculated glucose metabolic rate in GM (Fig. 5).

An exciting application of cGAN-aided motion correction is the possibility to address intraframe motion in static clinical PET scans. Clinical ^18^F-FDG brain scans are usually performed at times > 45 min with a typical duration of 10–20 min. Such relatively long frames are sometimes subject to considerable patient motion artifacts, which impair image resolution and reduce image contrast important for differential diagnosis. Our data suggest that due to the high tracer uptake in brain tissue at these late time points, these long frames can be partitioned into subframes as short as 15 s, which can be then processed with cGAN methodology to yield images of sufficient quality for accurate coregistration (Fig. 6). Thus, one can envision a reconstruction protocol in which list-mode data are sequentially divided into smaller and smaller subframes that are individually corrected for motion by taking advantage of enhanced image features generated by cGAN preprocessing, resulting in an overall improvement in image quality. We would like to point out that the short subframes (15 s) are only necessary to determine the exact time of displacement. Because realistic patient motion occurs in the form of a few distinct shifts in head position interspersed within a longer time frame, most motion vector parameters derived from the set of all short subframes will be negligible.

In this context, the question arises whether mappings are specific to one particular imaging system or whether they could be also applied to ^18^F-FDG data obtained from other imaging systems. Our very preliminary data suggest that mappings might be transferrable to other imaging systems on the basis of the application of our mappings to a dynamic ^18^F-FDG study (12 × 60 s, 4 × 120 s, 5 × 300 s) acquired using an external PET/CT system from a different vendor. The only requirement is that mappings should match their respective PET mid-times. Supplemental Figure 7 suggests that mappings might possibly be independent from the imaging system; however, this issue mandates further investigations that were not the focus of this work.

One of the main drawbacks of the study is the low number of test datasets. Although synthetic data with variable motion parameters were generated, they were still generated from the test datasets. Moreover, there are several other limitations that need to be considered when applying cGAN methodology in clinical applications. First, current implementations of cGAN processing are highly computationally intensive. The time to generate one (source-to-target) mapping pair is 17 h on a dedicated NVIDIA DGX Workstation with 1 × 32 GB Tesla V100 Volta GPU for a frame size of 344 × 344 × 127 voxels. However, once a mapping specific for a particular tracer is established via the training process, the time to apply this mapping to a low-count image of any individual subject is only 1 min. Another potential source of error might be intraframe motion in the reference frame. In this study, we did not correct for such motion artifacts because neither visual inspection of image quality nor close monitoring of the subjects during the last frame indicated noticeable subject motion. Finally, our cGAN implementation requires a training set that consists of image pairs that are devoid of motion artifacts. Because our data were acquired on a fully integrated PET/MR system, simultaneously acquired MR navigators were used to correct the training set for motion. When translating these findings to other sites, the use of cycle GANs (*33*), which produce generic mappings from spatially noncorresponding data, may be a potential solution that voids the requirement of motion-corrected image pairs.

## CONCLUSION

We present a data-driven motion-correction approach for dynamic ^18^F-FDG brain studies that is based on cGAN methodology. The method allows the derivation of an accurate motion vector for low-count frames as early as 2 min after injection, thus facilitating the derivation of an IDIF void of motion artifacts. The developed methodology has the potential to improve the accuracy of noninvasive absolute quantification in the context of clinical PET and PET/CT studies. In addition, cGAN methodology might also facilitate correction for intraframe motion, thus improving image quality of clinical scans of long duration.
